# Collaborative leadership and the implementation of community-based fall prevention initiatives: a multiple case study of public health practice within community groups

**DOI:** 10.1186/s12913-017-2089-3

**Published:** 2017-02-16

**Authors:** Maureen Markle-Reid, Cathy Dykeman, Jenny Ploeg, Caralyn Kelly Stradiotto, Angela Andrews, Susan Bonomo, Sarah Orr-Shaw, Niyati Salker

**Affiliations:** 1 School of Nursing, McMaster University, Hamilton, ON L8S 4K1 Canada; 2 Halton Region Health Department, Oakville, ON L6M 3L1 Canada; 3 Haliburton, Kawartha, Pine Ridge District Health Unit, Haliburton, ON K0M 1S0 Canada; 4 York Region Public Health, Vaughan, ON L4K 0G5 Canada; 5 Simcoe Muskoka District Health Unit, Barrie, ON L4M 6K9 Canada; 6 Brant County Health Unit, Brantford, ON N3R 1G7 Canada

**Keywords:** Collaborative leadership, Collective impact, Older adults, Fall prevention, Public health, Qualitative, Case study

## Abstract

**Background:**

Falls among community-dwelling older adults are a serious public health concern. While evidence-based fall prevention strategies are available, their effective implementation requires broad cross-sector coordination that is beyond the capacity of any single institution or organization. Community groups comprised of diverse stakeholders that include public health, care providers from the public and private sectors and citizen volunteers are working to deliver locally-based fall prevention. These groups are examples of collective impact and are important venues for public health professionals (PHPs) to deliver their mandate to work collaboratively towards achieving improved health outcomes. This study explores the process of community-based group work directed towards fall prevention, and it focuses particular attention on the collaborative leadership practices of PHPs, in order to advance understanding of the competencies required for collective impact.

**Methods:**

Four community groups, located in Ontario, Canada, were studied using an exploratory, retrospective, multiple case study design. The criteria for inclusion were presence of a PHP, a diverse membership and the completion of an initiative that fit within the scope of the World Health Organization Fall Prevention Model. Data were collected using interviews (*n* = 26), focus groups (*n* = 4), and documents. Cross-case synthesis was conducted by a collaborative team of researchers.

**Results:**

The community groups differed by membership, the role of the PHP and the type of fall prevention initiatives. Seven practice themes emerged: (1) tailoring to address context; (2) making connections; (3) enabling communication; (4) shaping a vision; (5) skill-building to mobilize and take action; (6) orchestrating people and projects; and (7) contributing information and experience. The value of recognized leadership competencies was underscored and the vital role of institutional supports was highlighted.

**Conclusion:**

To align stakeholders working towards fall prevention for community-dwelling older adults and establish a foundation for collective impact, public health professionals employed practices that reflected a collaborative leadership style. Looking ahead, public health professionals will want to shift their focus to evaluating the effectiveness of their group work within communities. They will also need to assess outcomes and evaluate whether the anticipated reductions in fall rates among community-dwelling older adults is being achieved.

## Background

Among community-dwelling older adults, falls are the leading cause of both fatal and nonfatal injuries [1]. While evidence-based strategies for fall prevention are available [2], persistent implementation barriers exist [3]. Falls are the result of the complex interaction between biological, behavioural, environmental and social risk factors, therefore they are challenging to address because there is no single solution or strategy that will apply to all circumstances [4]. Instead, experts advocate that the widest possible array of community stakeholders be mobilized to implement initiatives that target protective factors [5]. As these strategies must be delivered in various settings and across different time points [6], the goal is to reach beyond healthcare settings and engage the considerable number of individuals, professionals and organizations that are already working with older adults in community settings. One venue for this type of multi-sectoral collaboration is the community group that works jointly to undertake wellness programming and events, coordinated service delivery, policy or any other activity that aligns with the World Health Organization’s (WHO) “Active Ageing” framework for the prevention of falls in older age [4, 7–10]. These community groups, both through their organization and the processes they help to facilitate, are examples of collective impact.

Collective impact is an approach that Kania and Kramer (2011) describe as “the commitment of a group of important actors from different sectors to a common agenda for solving a specific social problem” [[11], p.36]. Using their language, we suggest that fall prevention is an example of an “adaptive problem”, a problem that is complex and whose solutions require resources and the influence of multiple entities in order to achieve the necessary changes [[11], p.39]. The collective impact approach is valuable because it identifies five conditions for success: a common agenda, shared measurement systems, mutually reinforcing activities, continuous communication, and backbone support organizations [11, 12]. Thus, for public health professionals (PHPs) who are participating actively in community-based fall prevention groups, and working to engage and align partners, collective impact is an approach that captures the problem’s complexity and proposes a pathway towards a solution that acknowledges the multifaceted and gradual nature of social change.

To work actively within community groups, individual PHPs are expected to demonstrate competencies that include collaboration and leadership [13]. PHPs need skills in team building, negotiation, conflict management, facilitation, and mediation, to enable them to work in partnership with others in pursuit of a common goal such as fall prevention [13]. Leadership can have many definitions and in the Canadian context the national agency responsible for public health defines this concept as “the ability of the individual to influence, motivate, and enable others to contribute towards the effectiveness and success of their community and/or organization in which they work”[[13], p. 12]. Further guidance for PHPs is included, notably the direction to inspire people, provide “mentoring, coaching and recognition,” and empower people so that “other leaders emerge” [[13], p. 12]. This terminology, with its focus on interactions, social processes and situated practices, draws upon conceptualizations of leadership that are more attentive to group dynamics and less leader-centric and attribute focused. Within this tradition, broadly identified as distributed, shared or dispersed leadership, is concept of collaborative leadership [14, 15].

The concept of collaborative leadership, similar to the other distributed or shared leadership forms, has certain characteristic properties: it emerges from a group of interacting individuals; it features open boundaries; and it encourages the distribution of expertise across many participants [14]. A key to understanding the particular concept of collaborative leadership, apart from other distributed styles, is the word “collaboration” which emphasizes the process of working together. A further feature is reciprocity of influence, and the manner in which collaborative leadership expresses a form of mutual influence. Significantly, Hallinger and Heck (2010) focus attention on the interaction of both socio-cultural processes (interactions between individuals) and structural processes (including features of the setting, institution or organization, for example) [16]. While a focus on what collaborative leadership is can bring valuable insight, the more appropriate focus for pragmatic investigations into individual practice, is what collaborative leadership and collaboration can do.

Advocates of collaborative practice have argued that this approach can effectively address complex issues, such as health promotion, and generate communal benefits, such as a sense of trust, ownership and synergy [17, 18]. Critics have charged that collaborative processes are slow, challenging to manage, and at risk of stalling due to inertia [19–21]. Furthermore, because collaborative partnerships can be difficult to measure and evaluate, their effectiveness is unclear [19, 21–23]. Despite these challenges, collaboration continues to be identified as a priority among funding agencies, foundations, and other stakeholder groups [20]. When considering fall prevention initiatives specifically, there are many evaluation studies [24–26]; however, there is little research on collaborative fall prevention initiatives within communities [27]. Thus, research is required to identify practical examples of collaborative practice and collaborative leadership, to inform practice and guide future evaluation of such initiatives within the community setting.

In this paper we distinguish between the structure (a community group), the process (collaboration or working together [28]), the PHP (an individual who can enact various leadership styles [29, 30] and the goal (engaging partners in order to deliver fall prevention strategies as defined within the WHO’s Active Ageing framework [4]. A stakeholder is defined as an individual or organization, either public or private, with an interest or concern in fall prevention.

Through Public Health Ontario’s Locally Driven Collaborative Projects (LDCP) program [31] (See: https://www.publichealthontario.ca/en/ServicesAndTools/LDCP/Pages/default.aspx), frontline PHPs collaborate with the research community to develop relevant projects that meet the needs of local communities. The program’s purpose is to foster partnerships, and produce relevant knowledge and transfer knowledge. This study of collaborative leadership practice originated with an LDCP project that investigated the readiness to implement fall prevention evidence. In a survey of health and non-health community service providers, the research team learned that 21% of providers reported having the knowledge and skills to implement the evidence, but only 10% felt they had the resources to support fall prevention activities [32]. While all providers reported a desire for future collaboration, favor was stronger among health sector providers (86%) than among providers from the non-health sector (56%) [32]. Recognizing that within public health, intersectoral collaboration is a valued strategy for encouraging resource and knowledge sharing, and that experienced practitioners are required to lead intersectoral collaboration [33], the research team developed a pragmatic research question: What collaborative leadership practices do public health professionals use to engage community partners in fall prevention initiatives for community-dwelling older adults?

## Methods

This was an exploratory, retrospective, multiple case study of four multi-stakeholder groups that worked collaboratively to implement fall prevention initiatives. The case study approach was appropriate because the research team’s intent was to obtain an in-depth understanding of groups and their practices. The use of multiple cases strengthens the results. Yin suggests we can learn from the similarities and differences among the cases and that greater confidence may be ascribed to the analytic conclusions that subsequently arise [34]. This study was exploratory because its aim was to understand how a contemporary activity is expressed in real world contexts. It was a retrospective study because participants were asked to recall their experiences. The advantages of this design are efficiency and the potential for generating participant insights that arise from reflection.

Researchers from ten public health units partnered with researchers from McMaster University to conduct this pragmatic and applied research study. Public Health Ontario provided funding and support through its LDCP program that was designed to build the research capacity of frontline PHPs through participatory learning and mentoring from experienced researchers [31]. To facilitate this learning process and enable full participation of geographically dispersed members, the research team employed a variety of strategies to enhance collaboration. The key strategies included the use of internet and telecommunication technologies and the use of readily available word processing tools (e.g. sorting and formatting functions) in lieu of the specialized and less accessible data management software. Mentoring in research methodology was delivered by team members who were senior investigators with expertise in qualitative methodology. Accessible and flexible formats were used, including: readings, teleconference presentations, planned assignments and ongoing feedback.

### Selection of cases

Public health professionals, who were members of the research team, identified potential cases by drawing upon their personal knowledge, making formal inquiries to their peers, and seeking the advice and recommendations of colleagues. Since the research question specifically addressed the practices of PHPs within Ontario-based community groups working to prevent falls, the groups being investigated included an actively participating PHP who delivered initiatives that aligned with the WHO’s Active Ageing framework [4]. To better understand the nature of group work, the team searched for groups with different membership compositions. By October 2013, 12 groups were identified and evaluated according to the inclusion and exclusion criteria (Table 1) in order to reach a short-list of five groups. To maximize learning, the short-listed groups featured traits that, when considered collectively, were diverse. Thus, when comparing the five groups, they had: (1) PHPs who were active but in different ways; (2) membership rosters that were more typical (comprised of agencies and organizations from the public and private sectors) and less typical (comprised of citizen volunteers); and (3) track records of completed initiatives that were varied in their structure, delivery and scope. The research team discussed the short-list and then identified the final four cases by reaching consensus. A maximum of four cases could be feasibly studied given the timeline and resources available.

**Table 1:** Case selection: inclusion and exclusion criteria

Criterion	Descriptor	Rationale
Inclusion criteria		
Public health role	Informal or formal leadership	Have potential to provide clear findings on public health’s collaborative leadership practices
Group membership	Diversity, specifically groups with healthcare professionals *and* others	Have potential to give context; to what extent is the potential of falls as everyone’s business realized
Initiative achieved	An initiative can be a policy, program, event or service that fits within the scope of the WHO Fall Prevention Model	Initiative may not be formally described as “falls prevention”; rather, it may be called Healthy Aging or Wellness, for example.
Exclusion criteria		
Initiative stalled or completed pre-2012	Seek groups that are actively planning, implementing or making improvements to their fall prevention initiative	The potential for accurate recall is strengthened
High membership turnover	Seek interview respondents and focus group members who can answer questions in depth	Case studies are looking for depth

### Recruitment of participants

Recruitment of participants for the focus groups and interviews was supported by the PHP who worked with the groups at the selected case sites. All group members were invited to participate in the research study via contact by e-mail and/or telephone. Participation was voluntary. Interested individuals arranged their participation by contacting the university-based research coordinator.

### Data collection

Using semi-structured interview and focus group guides, a trained interviewer asked respondents to describe: their role(s) and experiences in the group; the practice of working together; and the accomplishments and challenges they faced. Probing follow-up questions were used to elicit information about the specific practices used by the public health professionals. The guides were pre-tested with a local fall prevention group that was not included as a case site. To prepare for data collection, the research team asked each of the four groups to submit up to 15 documents, including a one-page story of the project. Of particular interest were documents that described each group’s processes for working together. In response to this request, the team received media releases, reports, presentations, meeting minutes and terms of reference.

The in-person focus groups captured the breadth of member perspectives and the interviews, held over the phone or in-person depending on respondent preference, provided an opportunity for exploring respondents’ experiences in depth. Some participants opted to participate in both data collection formats. Across the four case sites, 32 individuals participated: 16 in the focus group *and* the interview, ten in an interview only, and six in a focus group only. Participation by case site was as follows: Group A – six interviews and a focus group with six members; Group B - seven interviews and a focus group with six members; Group C – seven interviews and a focus group with four members; and Group D – six interviews and a focus group with six members. A breakdown of participation according to stakeholder role is provided in Table 2. Focus groups (*n* = 4) lasted approximately 90 min and the key informant interviews (*n* = 26) lasted approximately 60 min. All interview and focus groups were digitally recorded and then transcribed verbatim. Data collection started in June 2014 and was completed in September 2014.

**Table 2:** Study participants (*n* = 32)^a^ by network and membership role

Membership role	Community Network							
	A		B		C		D	
	Interviews *n* = 6	FG 6 members	Interview *n* = 7	FG 6 members	Interviews *n* = 7	FG 4 members	Interviews *n* = 6	FG 6 members
Public health professional	*n* = 3	*n* = 1	*n* = 1	*n* = 1	*n* = 2	*n* = 1	*n* = 1	*n* = 1
Community service provider	*n* = 2	*n* = 5	*n* = 1		*n* = 4	*n* = 2	*n* = 5	*n* = 5
Older adult	*n* = 1		*n* = 5	*n* = 5	*n* = 1	*n* = 1		

^a^ Sixteen participants attended the focus group and were interviewed: A (*n* = 3); B (*n* = 5); C (*n* = 4); D (*n* = 4)

### Data analysis

In the first stage of analysis, data from each case site were analyzed separately (within case analysis). Two analysts from the research team extracted data segments relevant to the research question and organized them into tables. Next, they used an inductive process to cluster quotations that were similar. Summary statements that described those clusters were also drafted at that time. The analysts clustered the data independently, with consensus reached later in the process. Once this phase was completed, the initial findings were shared with the entire research team so that all members could provide interpretive notes and advance the development of emerging categories. In teleconferences, the appropriateness of emerging categories was discussed. To conclude the within-case phase, the analysts prepared case summary documents, organized systematically and containing exemplar quotes. One of these documents was discussed and refined, using both large and small group formats, in a face-to-face team meeting held in January 2015. As the research team was geographically dispersed, the remaining three summaries were similarly assessed at later dates using e-mail and teleconferences.

The second stage of analysis involved a comparison of findings across the four case sites (cross-case synthesis) using the analytic technique that Yin [34] identified for use with multiple cases. The team collaborated to group categories together, develop generalizations and probe similarities about successful collaborative leadership practices across the cases. Guided discussions and the process of addressing team member feedback on early drafts of the research paper facilitated the process of developing “strong, plausible, and fair arguments” that were supported by the data [[34], p. 160].

Various strategies were used to enhance the trustworthiness of the findings, specifically credibility, transferability, dependability and confirmability [35]. Credibility, or how accurately the findings reflected participant experiences, was facilitated through transparent descriptions of the research process. The participation of experienced PHPs in the analytic process helped to ensure that the emerging themes considered the context of public health practice. Transferability, or the applicability of the findings to similar contexts or subjects, was facilitated by the multiple-case study design that featured diversity in cases and participants. Additionally, detailed accounts of the research process were provided to enable readers to make informed decisions about the applicability of the findings to their contexts. Triangulation of evidence helped the team reach conclusions that were more convincing and complete [34]. Dependability, or the consistency and quality of data collection and analysis, was facilitated through the following strategies: consistent procedures that were documented in minutes, correspondence and field notes [36]; formal searches for alternative, or rival, explanations [36]; and reflexivity exercises that reminded the research team about the need for broad thinking. The collaborative analysis process itself encouraged thoroughness. As Barbour (2001) stated, when multiple individuals are involved during analysis there is “the capacity to furnish alternative interpretations” [[37],p.1116] and, in this way, to temper any movements towards arbitrary or unsystematic conclusions. Finally, the words of participants themselves were used to illustrate findings.

### Ethics

This study was conducted in accordance with the Tri-Council Policy Statement: Ethical Conduct for Research Involving Humans [38]. Ethics approval was obtained from the McMaster University Research Board (#14-258) and was renewed yearly as required. All focus group and interview respondents provided written informed consent.

## Results

### Overview

Key characteristics of each case site are displayed in Table 3. In each site, a community-based fall prevention group implemented initiatives designed to enhance the health, participation, and security of older people [4] (e.g., wellness programming; community fairs; fall prevention clinics; enhanced communication mechanisms; modifications to the built environment through benches, stair ramps, snow clearing; advocacy, and educational workshops).

**Table 3:** Case characteristics

Case study	A	B	C	D
Initiatives achieved	• Community wellness fair featuring speakers and exhibitors • Fall prevention clinics • Co-hosting of a fall prevention workshop for health professionals • Networking for health professionals	• Temporary accessible ramp project • Assessment of the community’s age-friendliness (according to WHO criteria) via focus groups and a survey • Advocacy for age-friendly communities	• Community wellness fair • Mapping project to identify “hot spots” for high rates of falls. This informed the location choices for subsequent health, wellness and social events. • Learning series: topics focused on lifestyle and behaviours that impact bone health	• Operation of community gathering places that offer: social engagement; physical activity programming; health and wellness workshops; and group meal preparation.
Stage of development	Formed in 1994	Formed in 2008	Formed in 2005	Formed in 2010
Public health’s role	Historically chair, currently participant in group, and administrative support	Chair	Chair	Rotated as chair, and participant on steering committee
Public health professional	Health Promoter	Health Promoter	Health Promoter	Public Health Nurse
Community • Rural/Urban • % Older adult population (2011)	• Rural • 21%	• Rural • 22%	• Rural/urban • 15%	• Rural • 16%
Operational features • Frequency of meetings • Funding • Size of group	A coalition that functions as a working group of the core program • Monthly meetings • Initial funding from New Horizons for Seniors grant • 15 members on average per meeting	Membership of older adults across the region • Monthly meetings • Initial funding from New Horizons for Seniors grant • 8 members on average per meeting	A large group consisting of several subcommittees • Quarterly meetings • No funding received. Work is supported through contributions from participating members. • 16-26 members	Municipal staff provide administrative support • Bimonthly meetings • Funding from New Horizons for Seniors grant • 15 members
Membership	Service providers	Older adults	Service providers	Service providers

In three of the cases (Groups A, C and D), the members consisted primarily of community service providers, whose organizational mandates aligned with the goals of the individual groups. While individual providers cited a personal interest in promoting health and safety for older adults, participation in the group on behalf of their agency was valued because of the networking and information sharing opportunities their participation provided. In contrast, Group B was a committee comprised of older adults drawn together by personal interest or a sense of civic responsibility.

Within these groups, the PHPs had different formal and informal roles. In Groups B and C, where the PHPs occupied the formal position of meeting chair, each PHP had administrative responsibilities. Group B, notable because it was comprised of older adult volunteers, had a PHP who supported and actively encouraged the group throughout the implementation of their various initiatives. In Group C, with a membership drawn primarily from service providers, the support function provided by the PHP had a more technical or information-based quality. In Group D, the PHP did not occupy a formal position, but was an active participant who helped with grant applications and as an educator. In Group A, the PHP was a knowledgeable and active group member who worked closely with, and occasionally substituted for, the chair. The PHPs who were current and former chairs described a goal of sharing the chair position in order to promote equity and develop the group’s capacity. Achieving that goal was difficult because their fellow members either expressed a lack of interest or cited an inability due to other commitments or responsibilities.

During cross-case analysis seven public health professional practice themes emerged: (1) tailoring approaches to address context, (2) making connections, (3) enabling communication, (4) shaping the vision, (5) skill-building to mobilize and take action, (6) orchestrating people and projects, and (7) contributing information and experience. While these practice themes are described separately, each practice theme highlights aspects of how collaboration, or working together, is realized in actual practice.

### Themes

#### Tailoring approaches to address context

Tailoring referred to how projects and initiatives were adapted in scale and scope to be feasible. Feasibility addressed not only the local community context within which the fall prevention initiative was being implemented, but also the contextual characteristics of each group, such as their interests or abilities. All the groups were constrained by time, personnel and financial resources and all the groups wanted to ensure that their efforts would be appropriate for their communities. A member of Group C explained: “*it’s easier to maybe just start with some of these smaller initiatives and when capacity is generated and interest is built, maybe then move towards building a more sustainable program”* (CFG1). A member of Group B reiterated this sentiment by stating: “*when you refocus your telescope into the area of what’s possible and what can be done, you end up on this micro scale”* (B7). Similarly, a member of Group D explained: *“’Okay, [the idea to build a] wellness centre is not going to happen … but we have another really viable option that’s totally based on the needs of our seniors in our communities that we think we can build on and make work”* (D1).

When group members and PHPs discussed the context they also referred to the internal dynamics of the group itself. The group members varied in their ability or willingness to compete tasks, thus one public health professional stated, her “*biggest learning*” came from understanding the group and *“what they’re capable of, whether or not they need to change or move, or if they’re okay just being where they are*“ (A5). Participants explained that *“well it’s too hard … you know I can’t be involved every month”* (C2) or “*I just didn’t want to do that sort of thing*” (B4). Tailoring for context also meant paying attention to community dynamics. To be effective, groups had to be “*socially careful*” (B4) and display sensitivity to other organizations over contextual issues such as their “turf” when it came to their program or organizational mandates. For Group A, this was a concern that the group’s community wellness fair could potentially detract from other existing senior-focused events.

#### Making connections

The practice of making connections was focused on how relationships were forged between people, things and ideas. For each of the groups, forging connections with “*higher up folks*” and “*other organizations that are trying to achieve things that are similar”* was an essential component of their efforts to align priorities and initiatives in order to promote efficiency and reduce duplication (A5). The practice of making connections was described using the metaphors “*bridge*” and “*link*”(A6) or in phrases such as “*reaching out”* (B2). In all instances, the PHPs described a purposeful process where they facilitated relationship building. From the perspective of their fellow group members, public health professionals were ideally suited to take on this role. As one community service provider explained: “*what public health can do is … allow their staff to be out in the community and be in groups like this [and] be involved”* because with its “*structure*” and “*broad*” scope, public health can *“pull programs together”* (D3).

For the PHPs, however, the practice of making connections applied to more than just connecting with people, but also connecting ideas. The idea that initiatives promoting age-friendly communities or focusing on “*those healthy living components*” (C5) could actually be considered fall prevention was new learning for many group members. Describing the “*input that public health gave us,*” one group member recalled: *“the key learnings for me”* were about *“positive messaging”* because *“I didn’t really know that falls … was so laden with negative connotations for older adults”*; it was something *“I didn’t learn … in nursing school”* (C7).

#### Enabling communication

The practice of enabling communication was described as “*keeping the lines of communication open*” and “*feed[ing] information back and forth*” (C1). As a process, collaboration required that all group members be open to ideas from their peers, thus a continual bi-directional flow of communication was perceived as essential. This type of communication required ongoing effort, and as one PHP explained: “*I do think that communication is one of the areas that you have to continually work on and come up with new and creative ways to keep everyone in the loop”* (A6). In practice, this meant developing relationships, asking partners what support they needed, and taking the time to “*do that real check-in*”(A6). The importance of communication was emphasized by its formal inclusion in each group’s Terms of Reference. Communication both within and beyond the group was framed as member’s “*responsibility*” and “*duty*” (Case D, Terms of Reference) and a basic member requirement (Group A, Terms of Reference). Under the heading “Activity Parameters,” case D’s Terms of Reference stated:

*…information sharing is fundamental to community-based planning … the [group] will facilitate formal and informal information exchange, provide opportunities for programs to present and seek information and ideas while jointly exploring new approaches and best practices. This will include efforts to inform and encourage the public about what is available and how to access it.* (Case D, *Terms of Reference*)

#### Shaping the vision

A key practice enacted by the PHP as a member of the different groups was developing and communicating a vision that shaped all aspects of the group’s work. This visioning practice aligned with public health’s mandate to focus on broadly based community health promotion to achieve injury prevention. For these groups whose initiatives focused on fall prevention, the vision was “*falls prevention really includes healthy living*” (C5) and that “*there isn’t one sector that’s going to be able to do it alone. There isn’t one organization that’s going to be able to make it happen. So it is going to be that collaborative partnership”* (A6). A group’s vision, or purpose, was typically formalized through the Terms of Reference. In collaborative group settings, the presence of a clear, formal statement of purpose was valuable. In the words of one community service provider, the group does not act simply because it was “*somebody’s idea*”; rather the impetus for action “*actually*” came from the Terms of Reference and “*we have to accept it.*” Thus, with clear objectives, decision-making becomes “*simple*” (C2). Vision shaped everyday practice because it informed the identification of the problem and potential strategies to address the problem. Thus, PHPs worked to emphasize evidence-based solutions to address fall prevention by employing specific strategies: leveraging their expertise and/or formal role as chair or co-chair to identify agenda items, re-direct off-topic discussions and share information (data, best practice, etc.).

#### Skill-building to mobilize and take action

A key goal of the PHP was to build capacity within the group to enhance the groups’ effectiveness and sustainability in delivering fall prevention within the community. According to one PHP, “*I have to build [the] capacity of others … so that it’s just not me*” (A6). The word “capacity” involved developing collaborative teamwork abilities and administrative or organizational skills. To develop that capacity and mobilize their groups, PHPs assessed their groups and helped to identify each member’s talents and abilities because *“…everybody plays a role, and everybody’s role is really important, but what we need to ensure is that … we can build on the strengths of each person’s role or each organization’s role to [prevent a fall]”* (A6). Building an effective group also required that PHPs complete a “*gap analysis*” of who was engaged and who still needed to be engaged (A6). To fill gaps, recruitment or mentoring was conducted. In group C, the PHP encouraged a member with strong interests and expertise in older adult fitness to form a subcommittee. Describing this welcomed mentorship, the participant stated: “*not only do I get support, but I also get gentle help”* (CFG6).

#### Orchestrating people and projects

As a practice theme, orchestration referred to management of people and projects. Often, the responsibility for organizing meetings and operating projects was the responsibility of the PHP (Groups A, B, C), or a group member who was affiliated with a different public institution (e.g., the district government in Group D). All of the groups required someone to take responsibility for scheduling, taking minutes, distributing meeting materials and arranging space for meetings or events. PHPs were perceived by their fellow group members as being ideally suited for organizational and administrative work because they had technical and administrative skills, and could facilitate access to key resources. They were in a position to make participation “easy”:

*And then to bring out the best in them, you have to be organized. Don’t make their life difficult, make it easy. And I’m not necessarily talking spoon feeding, but I’m talking do whatever you can to make their life easier. Like I always organize minutes and I organized agendas and I would have copies and I would send these things out, so people didn’t have to scramble and look and having the meetings at the same time. They didn’t have to figure out now is it this Tuesday or next Thursday. Like the more you can do to make their lives easier so that they can put their energy into the real work.* (A4)

Orchestration also encompassed efforts to promote a welcoming culture and inclusive feel. In practice, this was realized by prioritizing collaborative and shared decision-making. Respondents from all four groups described their environments as ones where there were opportunities for input, discussion and dissent. Statements like *“everybody’s opinions are valued and listened to which is wonderful*” (D1) or “*I can stick up my hand and disagree with somebody … it’s just part of the discussion*” (D3) were found in all groups. Nevertheless, some personality conflicts and disagreements occurred and there were perceptions that decision making power was unevenly distributed (e.g. Groups B and D). While the PHPs described their efforts to support a positive culture, there was attrition in Groups A, B and D due, in part, to differing expectations about the group and its processes.

#### Contributing information and experience

As each group worked towards its goal of promoting fall prevention, the PHPs supported their group by bringing forward specialized information, including details about evidence-based fall prevention strategies. Their expertise, experience and access to institutional resources supported this process. In Group C, the PHP shared information about fall “hot spots” in the community; places where there were higher than average rates of falls among the older adult population. One community service provider, recalling the presentation, stated that she had “*hunches*” about these trends, but the PHP provided the evidence (C7). Thus, in the words of the PHP, the event “*didn’t just happen*” out of “*nowhere*”; rather it was based on a “*very good background of information*” (C5). Among the other groups, PHPs drew upon their expertise in community assessment (A and C), grant writing (D) and guiding the group’s accessible community spaces project through their locality’s building approval process (B). Group members valued this sharing of information and expertise because PHPs were seen to be credible and their credibility enhanced the group’s efforts. This sentiment was expressed directly: *“…public health has respect in the community, so … when we’re backed by public health I feel like that gives a lot of credibility in the community”* (AFG3).

## Discussion

To our knowledge, this is the first study to explore how public health works together with community groups, employing a collaborative leadership style, to collectively implement community-based fall prevention initiatives for older adults. While the groups at the four case sites delivered a range of valuable initiatives, there was little evidence of pre-implementation needs assessments or of plans to evaluate the fall prevention initiatives. Collaboration, promoted and valued within these groups because of its potential to foster innovative solutions, was reported. Overall, the goal was collective impact, or the incremental development of momentum towards social change. Were the collaboratively delivered initiatives described here innovative and did they achieve collective impact? The answer depends on perspective. In one respect, programs and events were introduced into communities where they did not exist before. Arguably, each initiative was innovative and worthwhile for the community being served. Kania and Kramer (2011) argue that collective impact is slow to achieve, and difficult to measure [11]. For the groups studied, a key achievement was establishing a foundation for social change. In all cases, engagement occurred. The next step will be to evaluate the group processes for sustainability, and to understand the impact of the seven collaborative leadership practices that were identified through this study. Longer term, each group will have to evaluate whether they achieved their Active Aging goals and achieved a reduction in the rates of falls and fall-related injuries and death.

The collective impact approach has pragmatic value because it captures what both the PHPs and the community groups sought to accomplish. To understand this further, it is helpful to consider the way that the seven practice themes align with Kania and Kramer’s five conditions for collective impact. First, is the necessity for a common agenda which was achieved with the common focus on the WHO’s Active Aging pathway to fall prevention [4]. Second, is the shared measurement system. To the extent that a fall prevention initiative was defined by the common agenda, it was possible to ensure aligned efforts; however, this is only a first step. Shared measurement systems must also address accountability and this was the piece that was largely missing from the cases included in this study. A third element of collective impact is the presence of mutually reinforcing activities. The emphasis on coordination, whether through practices of tailoring, orchestrating or connecting was evident. The fourth element of collective impact, the existence of a background support organization, was perhaps the most significant. In this study, the support of the public health agencies (Case Sites A, B, and C) and a district government (Case Site D) organization was essential to the operation of the fall prevention groups. Nowell and Harrison (2010) state that for group work to move forward, passion for an idea can forge a common cause, but there is a “tipping point” where group effectiveness is achieved because a combination of “institutional legitimacy, organizational capacity, and political capital” exists [[39], p33]. Finally, collective impact requires continuous communication, which was demonstrated in all four cases.

The literature on collective impact suggests that when community partners align, the impact on delivery will be improved coordination and outcomes. Ultimately, further research will be required to establish whether this actually occurred in the settings we studied. There will be challenges in accomplishing this goal of evaluating impact because collective impact is slowly realized [11, 12]. Moreover, theoretical work will be required to better conceptualize the nature of the relationships (i.e., causal, mediated, etc.) between the adoption of collective impact and the delivery of desired public health outcomes. The most important consideration is to understand that the collective approach represents a value stance. While the evidence to support its effectiveness is inconclusive, the approach aligns with the principles of democracy and equity that are highly valued by policy makers and funders within the health and community care sectors.

Collective impact hinges on collaboration. The process of working together is heralded as a means to achieve optimum health outcomes for vulnerable populations [8, 40, 41]. Its status as social good is evidenced by the fact that health dollars are increasingly being directed towards collaborative partnerships [18, 42]. But, are community groups the best way for public health to achieve collective impact? Some researchers suggest that more evidence is required before it can be stated that there is a true return on investment with this approach [43]. Evidence for effectiveness is inconclusive and even when public health professionals are working across boundaries with their collaborative partnerships, the tendency towards silos persists, particularly in larger networks [44]. The benefits of trust and more resources tend to flow more towards groups that are highly connected and comprised of members with strongly aligned mandates and goals, while the more diverse groups with broader membership tend to experience challenges [45]. The reasons for working together are well understood: it is valued; it is a policy imperative; and it brings methodological advantage.

Scholars studying collaborative leadership state that large-scale effects such as changes to a population’s health are often unrealistic and that evaluators are better advised to consider intermediate outcomes [19, 46]. Even if collaboration only imparts the most basic of benefits, namely information sharing and a parallel breaching of institutional silos, community members benefit because of the potential for better service coordination [46]. Likewise, researchers who have studied diversity note that collaboration is challenging; success requires varied and sustained supports (e.g. transportation, child-care, training, mentoring etc.) [46, 47].

### Limitations

There were potential sources of bias that resulted from the study’s design. Purposeful sampling of the case sites was carried out by PHPs who identified groups from among their peers. While there were formal sampling criteria that emphasized diversity, there was a subjective element that must be acknowledged. As well, by selecting groups that had successfully implemented initiatives, we potentially excluded the insights that may have emerged from groups whose initiatives had more challenges. Second, most of the case study data was generated from focus groups and interviews and was subject to recall bias. Emerging themes were corroborated by having multiple cases, and multiple respondents within each case. While this study did not include observational data, it is a strategy for future researchers to consider. Finally, the sample of case sites included three rural and one suburban site. This must be acknowledged because it is reasonable to expect that different styles of leadership may be required for groups drawing upon and serving culturally and economically populations that are more typical of urban areas. This topic will also be an area of interest for future research.

### Future research

A comparison of engagement outcomes, depending on combinations of different stakeholders in the groups was not conducted due to the limited number of case sites and the scope of research question. However, groups that include various stakeholders (community service providers versus healthcare providers versus older adults) in different degrees of representation could have different experiences. Potentially, the direction, level of collaboration and types of initiatives generated could be impacted by particular membership mixes. A collaborative group with higher representation from older adults could potentially drive the development of initiatives that directly impact older adults (including their access to services, events or outreach programs for their own demographic, etc.). This study did not focus on differential outcomes based on the role composition, but it could be a topic for future research teams to consider.

## Conclusion

Public health professionals, with their mandate and scope of practice, are ideally suited to lead community partnerships charged with implementing fall prevention initiatives. Examination of four cases, purposely selected because of the active role played by a PHP, highlighted the leadership potential of these individuals. The seven practice themes were reflective of a collaborative leadership style, As implemented by public health, collaborative leadership practice demanded specific efforts from each professional (tailoring for context, communicating, connecting, visioning and skill building) and drew upon the institution’s supports and skills (orchestration and technical contributions). These efforts established a foundation for collective impact and demonstrated a purposeful approach towards addressing fall-prevention; one that incorporated a recognition that solutions must be delivered through the coordinated and sustained efforts of multiple stakeholders. Looking ahead, PHPs will want to focus on evaluations of effectiveness, not only for the fall prevention interventions they design and implement, but also for the collaborative practices they increasingly employ.

## Abbreviations

A [#]: Case (or Group) A, Member #
AFG [#]: Case (or Group) A, Focus Group, Member #
B [#]: Case (or Group) B, Member #
BFG [#]: Case (or Group) B, Focus Group, Member #
C [#]: Case (or Group) C, Member #
CFG [#]: Case (or Group) C, Focus Group, Member #
D [#]: Case (or Group) D, Member #
DFG [#]: Case (or Group) D, Focus Group, Member #
FG: Focus Group
LDCP: Locally Driven Collaborative Project
PHO: Public Health Ontario
PHP: Public health professional
WHO: World Health Organization
